# Tick fauna of Malaysian red jungle fowl (*Gallus gallus*) in Bangi, Malaysia

**DOI:** 10.14202/vetworld.2015.1167-1171

**Published:** 2015-10-09

**Authors:** M. Konto, G. I. Fufa, A. Zakaria, S. M. Tukur, M. Watanabe, S. D. Ola-Fadunsin, M. S. Khan, Y. M. Shettima, S. M. A. Babjee

**Affiliations:** 1 Department of Companion Animal Medicine and Surgery, Faculty of Veterinary Medicine, Universiti Putra Malaysia, Serdang, Malaysia; 2 Department of Veterinary Laboratory Diagnostics, Faculty of Veterinary Medicine, Universiti Putra Malaysia, Serdang, Malaysia; 3 Department of Microbiology and Parasitology, Faculty of Veterinary Medicine, University of Maiduguri, Nigeria; 4 Department of Bio Sciences, Gomal College of Veterinary Sciences, Gomal University, Dera Ismail Khan, Pakistan

**Keywords:** Bangi, Malaysia, red jungle fowl, tick fauna

## Abstract

**Aim:**

The red jungle fowl is generally considered as one of the endangered Asian wild Galleopheasants due to man-made encroachment of their habitats, coupled with the effect of disease and disease causing organisms like ticks and tick-borne infections. This study aimed to determine the tick fauna of the red jungle fowl and their predilection sites based on developmental stages.

**Materials and Methods:**

A total of 33 jungle fowls were sampled for this study from Bangi area of Selangor State, Peninsular Malaysian. The birds were captured using a locally made trap made-up of loops and bites. Ticks present on their bodies were detached using fine forceps and identified morphologically under a dissecting microscope.

**Results:**

91% of the jungle fowls were infested with ticks, all of which belongs to the species *Haemaphysalis wellingtoni*. The ear region appeared to be the most common predilection site (63%) for all the developmental stages in which the larval stages are solely restricted to that region. Nymphal and adult stages were distributed on the comb, wattle, and facial region in addition to the ear region.

**Conclusion:**

This study was the first in its kind and showed a high prevalence of tick infestation among jungle fowls. *H. wellingtoni* was known to be a vector in transmission of many tick-borne pathogens. Therefore, there is the need for further investigation to identify the various pathogens associated with this tick.

## Introduction

Ticks are one of the most important ectoparasites of red jungle fowl. They are found attached to different parts of the body sucking blood and increasing the risk of infection to various microorganisms in humans and animals [1-3]. Clinically, they can be a nuisance; their bites can cause irritation and itching, accompanied by tissue and humoral reaction of the host, hyperemia, eosinophil infiltration, and a local edematous reaction [4]. The damaged tissues are pulled by the weight of the feeding tick and thus produce a sensation of pain [5-7].

**Figure-1:**
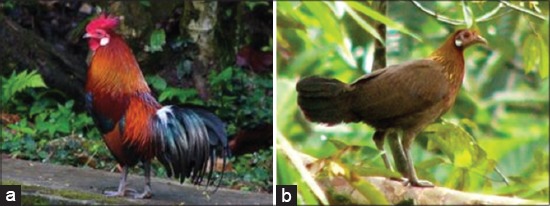
(a) Male *Gallus gallus gallus*, (b) female *G. gallus gallus*.

**Figure-2:**
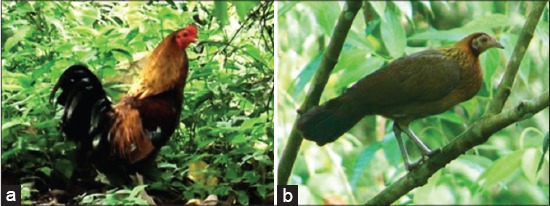
(a) Male *Gallus gallus spadiceus*, (b) female *G. gallus spadiceus*.

Among the four species: Red jungle fowl (*Gallus gallus*), Sri Lanka jungle fowl (*Gallus lafayetii*), Grey jungle fowl (*Gallus sonneratii*), and Green jungle fowl (*Gallus varius*), the red jungle fowl was considered of more historic importance for being the likely ancestral origin of our domestic chickens [8-10]. Two subspecies of the red jungle fowl are *Gallus gallus gallus* and *Gallus gallus spadiceus*. An important distinguishing feature between the two is the presence of a white ear patch in the subspecies *G.g. gallus* (Figure-1a and b) while the subspecies *G.g. spadiceus* has a red ear patch (Figure-2a and -b). They are cosmopolitan in distribution and more common to the foot of the Himalayas (Northeast India), southern China and down to the Southeast Asian region [8,10]. One distinctive characteristic of this species as compared to other galleopheasants family is that the male does not take part in the incubation of eggs or rearing of the younger ones [11].

Tick infestation among this class of birds is highly under estimated, leading to a paucity of information worldwide. This research was, therefore, designed to study the tick fauna of Malaysian jungle fowls.

## Materials and Methods

### Ethical approval

This study was conducted in Universiti Putra Malaysia under the research grant No. 9424000. Informed consent and approval was obtained from the communities where sampling was conducted and assurance of anonymity, prior to sampling. Procedures were conducted in such a way that cruelty to the animals was minimized to the minimum.

### Study area

**Figure-3:**
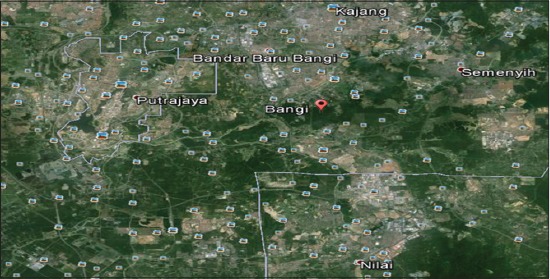
Photomicrograph of Bangi (sampling location).

Bangi (Figure-3) was until recently considered as a rural part of Selangor State, covered by oil palm plantations which is under the Hulu Langat district, southeast of Selangor State in Malaysia. Located between Latitude 2°55′14.54″ N and Longitude 101°46′50.98″ E with a range of 9235 m, it borders Kajang to the North, Semenyih to the east, Nilai to the south, and Putrajaya (Malaysia’s administrative capital) to the west. The area is sighted for Golf Resort, Hotel for visitors, and some residential neighborhood in Kajang. The area has an average relative humidity of 83%, temperature ranges between 33°C and 23°C, precipitation of 4 mm and an average visibility of 5 km all year round with slight variation.

### Sampling methods

A total of 33 jungle fowls from both sexes and age groups (young and adult) were sampled for the presence of ticks and their predilection sites based on developmental stages of the ticks collected. The birds were captured using a locally made trap, made-up of loops and bites which were set by fixing it onto the ground in such a way that the birds will not easily detect it. The birds were then systematically directed toward the trap and eventually got trapped from the leg. Captured birds were gently released from the traps manually, examined for the presence of ticks and later tag and released to the wild unhurt.

### Collection of ticks and identification

The jungle fowls caught were physically examined for presence of ticks on their body; ticks present were detached using fine forceps, carefully done to avoid destroying the mouthparts and were preserved in absolute alcohol before transporting them to the laboratory for identification.

Ticks collected from individual birds were put into Petri dishes according to their predilection sites and examined under a dissecting microscope and identified using the classification keys of Audy [12]. Furthermore, individual ticks were mounted as male and female, on dorsal and ventral views to identify their species using taxonomic characteristics such as shape and length of mouthparts, punctations and ornations, presence of leg bands, scutum color, presence or absence of festoons, and shape of the basis capitulum [12].

### Statistical analysis

Data were entered into Microsoft Office Excel spread sheet, and statistical analyzes were performed using Statistical Package for the Social Sciences version 21.0 (SPSS Inc., Chicago, IL). Relations between categorical outcomes were compared using the Chi-square test and the Fisher’s exact test where sample sizes were small. Differences between predilection sites and developmental stages were analyzed using one-way analysis of variance and Turkey *post-hoc* test. Statistical significance was set at p≤0.1.

### Results

**Figure-4:**
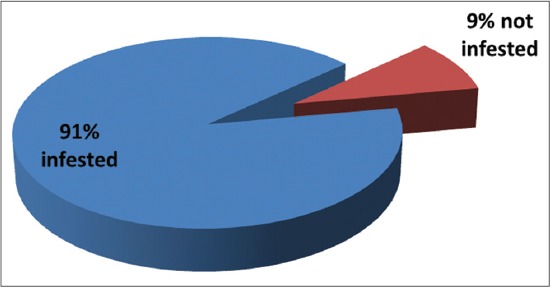
Prevalence of tick infestation in the red jungle fowl.

**Figure-5:**
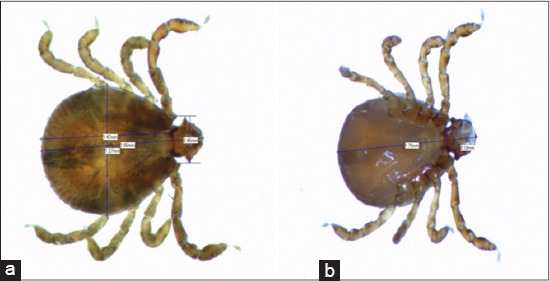
(a) Dorsal view of *Haemaphysalis wellingtoni*, (b) ventral view of *H. wellingtoni*.

The results of this investigation revealed that 91% of the jungle fowls sampled were infested with hard ticks while only 9% were not tick infested (Figure-4). Following identification, all ticks found belong to the genus *Haemophysalis* spp. (Figure-5a and -b) with body length and width ranging between 1.95-1.69 and 1.53-1.36 mm, respectively, in adult male (at ×40 optical magnification). Side by side length of the first two palpi that projects laterally from the basis capituli ranges between 0.48 and 0.42 mm (at ×40 magnification) in adult males.

Prevalence and distribution of ticks on the jungle fowls based on sex and age showed that both male and female fowl’s harbored ticks at different predilection sites. Both young and adult birds were also tick infested. Infestation rates in both sex and age groups were not significantly (p>0.1) different. The ear appeared to be the most common predilection sites among the fowls being sampled (Table-1).

**Table-1:** The prevalence and distribution of ticks on the jungle fowls based on sex and age.

Category	Animals infected (n=30)	Predilection site (%)			

		Comb	Ear	Wattle	Face
Sex					
Male	6	2	4	4	-
Female	24	4	18	10	2
Age					
Young	2	-	2	-	-
Adult	28	6	18	14	-

Distribution of ticks based on developmental stages during their life cycles showed that out of the total number of ticks collected, the ear predominate with 63% as the most predominant (p<0.1) predilection site for all the developmental stage (Table-2). The larval stage was solely restricted to the ear while the adult males were more commonly found on the wattle. There was no significant difference (p>0.1) between the developmental stages (Table-2).

**Table-2:** The distribution of ticks based on developmental stages of their lifecycle.

Category	Number of ticks collected (%)	Predilection sites (%)			

		Comb	Ear	Wattle	Face
Total number of ticks	188 (100)	24 (13)^a^	118 (63)^b^	44 (23)^a^	2 (1)^a^
Developmental stage					
Adult	66 (35)				
Male	32 (17)	4	6	28	-
Female	34 (18)	4	20	10	-
Nymph	50 (27)	16	26	6	2
Larvae	72 (38)	-	62	-	-

Row values with different superscripts for total number of ticks collected between the predilection sites differs significantly with others (p<0.1)

## Discussion

Susceptibility to tick infestation among jungle fowls as seen in this study agrees with an earlier report [13]. However, the previous report did not give information regarding the prevalence and distribution of the various developmental stages to their most preferred predilection sites. Susceptibility to tick infestation, other ectoparasites and associated pathogens among Avian spp. has been related to their feeding habit which is scavenging throughout the environment [13-15]. Out of curiosity, about 200 village chickens were also screened for the presence of tick infestation in the same locality; and the result of our investigation revealed that none of them was found to be tick infested. This finding agrees with earlier reports from India [7]; however, the actual reason to that finding was largely unknown.

The dominance of *H. wellingtoni* as the most prevalent tick species on jungle fowl in the area and the exclusion of some soft ticks like *Ornithodorus* spp. might be due to the fact that the sampling was conducted during the day time in which some nocturnal tick vectors like *Ornithodorus* spp. were unable to be detected [16]. However, the presence of *H. wellingtoni* spp. on the jungle fowls supports an earlier report [12] who stated that the domestic fowls are classified hosts to *Hemaphysalis bispinosa* *and H. wellingtoni* in Malaya (Peninsular Malaysia) and Borneo and Malaya (Peninsular Malaysia), respectively. However, there is a possibility that these vectors were transferred from the wild to the domestic variety, since a previous unpublished study by Amin-Babjee and Lee in 1994 has shown the presence of *H. wellingtoni* in the area.

*Haemophysalis* spp. have been reported to serve as vectors for the transmission of many disease causing agents in man and other animals from different parts of Asia, some of which are of bacterial origin causing Lyme borreliosis in China [17,18], viral encephalitis [19], parasitic diseases such as bovine theileriosis in Australia [20,21] and different kinds of rickettsia diseases from the Mediterranean [22], far east Asia [23,24] to Australia [25].

The number of ticks collected is spread across both sex and age as a result of intimacy between the opposite sexes, more especially during courtship. Another source of infestation is when the jungle fowls fall sick and hurdled themselves and stand still waiting for scavenger ticks. Transmission of infestation between young and adults fowls may be due to intimacy between older ones and the young ones. Similar findings have been reported in other mammals [1,26,27].

The ear region is the most dominant predilection site because of the important role it plays in nourishing the engorged female with blood meal from its numerous superficial micro-capillaries that enables the tick to suck blood. It also provides a favorable environment that aids the engorged female and its eggs or larval offspring from detrimental effects of the external environment. It provides optimum temperature for growth, development, and proliferation of the different developmental stages. The adult male tick found in the ear as seen in this study are newly metamorphosed nymphs to adults. This can easily be observed and differentiated by body size.

## Conclusion

It can be concluded that *H. wellingtoni* is the common tick species found on Malaysian red jungle fowl in the study area, and the ear is the most dominant predilection site for most of the developmental stages. The tick species identified and its high prevalence in the study area calls for the need to curb their menace, as this species is known to transmit different pathogens some of which are of public health significance.

## Recommendations

This study covers only a small unit area and uses a small sample size; therefore, it is recommended that a larger sample size with many sampling units should be used and the need to further investigate associated tick-borne pathogens is paramount.

## Authors’ Contributions

SMAB and MK conceived the project. GIF, AZ, SMT, MW, SDO, MSK and YMS participated in the general design, sample collection, data analysis, draft and revision of the manuscript. All authors read and approved the final manuscript.
